# Annotations

**Published:** 1905-06-03

**Authors:** 


### ANNOTATIONS.

### The Physical State of the British Nation.

A movement of considerable interest and importance will be inaugurated at a meeting which is to be held at the Mansion House on the afternoon of Wednesday, June 28th, under the presidency of the Lord Mayor. The speakers will include the Bishop of Ripon, the Lord Chief Justice, Sir William Broadbent, Sir James Crichton Browne, Mrs. Bramwell Booth, Alderman Strong and Mr. Compton Rickett, M.P., but the cause which they are to advocate is also assured of the support of a long list of distinguished personages who have consented to act as vice-presidents of the proposed National League for Physical Education and Improvement. The objects of the League are to stimulate public interest in the various societies which make the improvement of the physical condition of the people their special care; to bring into close association, and, if possible, to federate such societies, so that they may have increased strength—financial and otherwise; to encourage and advise the societies which find it difficult to hold their own under present conditions; and to render assistance towards the starting of organisations for the benefit of physical health and well-being wherever they do not already exist, as, for instance, in remote or poor districts. Nearly a score of eminent medical men, four archbishops, and several other high ecclesiastics, more than a dozen well-known lawyers, as well as a number of authorities on education and science, popular peers and athletes have identified themselves with the movement, which, we believe, owes its inception to Mr. Francis Galton. We sincerely hope that this galaxy of talent will be able, not only to bring home to the public mind the fact that the physical state of the British nation is far from satisfactory, but will also succeed in accomplishing some practical reforms. The first step towards this end will, we presume, be the collection of reliable data showing clearly in what directions there are grounds for apprehension concerning the coming race.

### Prevention of Infantile Mortality.

Another conference which, equally with the Mansion House meeting mentioned above, shows how tender the public conscience has become towards matters which affect the welfare of our race, will be held at the St. Pancras Town Hall on Monday. The specific objects which the conference is intended to promote are the cultivation of the health of prospective mothers, so as to strengthen their offspring, and to prepare the mothers to nurse their infants when born; to encourage breast-feeding of infants during the first nine months of life; to assist mothers to retain the best conditions of health during the period of lactation; to reduce the number of hand-fed infants to the smallest possible proportion; and to devise means for providing the purest and freshest milk for infants who cannot be fed at the mother's breast. Already the public health authorities of St. Pancras have started machinery which is likely to go some way towards the attainment of these ends. Last year they framed and put into practice a scheme whereby a complete list of the birth returns is furnished every week from the district registration offices. To the address of every mother whose name is included in the weekly returns a card of advice is immediately dispatched. In addition to this, the women inspectors select from the weekly returns cases which seem suitable to visit, and call at the mothers' homes to make inquiries, to give general information in the hygiene of nursing, and to direct the necessitous poor where to apply for such advice and assistance as may be urgently required. Experience has already shown that in St. Pancras, as in other places where women inspectors are employed, the poor do not as a rule regard such a visit as an unwelcome intrusion, but are often grateful for the kindly help and instruction that are offered to them. The arrangement may therefore be regarded as a great success. It remains now to devise a means of obtaining the purest and freshest milk for those infants who must be fed by artificial means. We shall be interested to learn what the opinion of the conference will be on this matter. We have more than once pointed out the drawbacks of the "infants' milk depots," and we hope that on this occasion the consensus of opinion will be in favour of preventing contamination of milk, rather than the partial or complete sterilisation of an already filthy supply.

### Leprosy and Isolation.

In a letter to the *Times* Mr. Jonathan Hutchinson intimates the reception of a petition from a number of Boers praying him to use his influence to draw attention to the compulsory deportation of some of their relatives to Robben Island. The signatories plead that if isolation is deemed necessary, it might at least be carried out near their homes, so that immediate relatives may have opportunities of knowing the condition of those dear to them. The present arrangement, assuming that the statements of the Boer petitioners are accurate, would be paralleled were a patient suffering from tuberculosis in Bristol to be banished compulsorily for life to one of the smallest of the Orkney Islands, and it is not necessary to suggest the outcry which would follow any such procedure in this country. Even those who hold most strongly to the doctrine of contagion do not place the danger as one of great urgency and Mr. Hutchinson quotes Dr. Hansen, whose advocacy of segregation is well known, as saying "we have (in Norway) no particular rules for the transport of patients. I see no reason for them. Leprosy is a disease of which it is not necessary to have any particular dread," this last sentence of course referring solely to the risk of contagion. Certainly it will be generally admitted that even if isolation be necessary, it should be carried out with a minimum of harshness. And we should like to ask why the lepers referred to in Mr. Hutchinson's letter were not sent to the leper asylum near Pretoria, which has been employed for the segregation of Transvaal lepers for many years past. If Robben Island was selected because it is not less accessible than Pretoria for the relatives in the case under consideration, that is merely a proof that additional leper asylums are urgently needed in South Africa.

